# Supplementary material for: A Critical View of the Application of the APEX Software (Aqueous Photochemistry of Environmentally-Occurring Xenobiotics) to Predict Photoreaction Kinetics in Surface Freshwaters
Source: Molecules. 2019 Dec 18;25(1):9. doi: 10.3390/molecules25010009 (PMC7017383; doi:10.3390/molecules25010009)
Supplement: Supplementary file 1 [file molecules-25-00009-s001.zip › 3.2.4_gcc-4.4.0.zip!/3.2.4_gcc-4.4.0/3.2.4_gcc-4.4.0/doc/octave/html/interpreter/Bug-Lists.html]

Bug Lists - Untitled


Next: Bug Reporting,
Previous: Bug Criteria,
Up: Trouble


---

### E.4 Where to Report Bugs

If you have Octave working at all, the easiest way to prepare a complete
bug report is to use the Octave function `bug_report`. When you
execute this function, Octave will prompt you for a subject and then
invoke the editor on a file that already contains all the configuration
information. When you exit the editor, Octave will mail the bug report
for you.

If for some reason you cannot use Octave's `bug_report` function,
send bug reports for Octave to bug@octave.org.

**Do not send bug reports to** help-octave. Most users of
Octave do not want to receive bug reports. Those that do have asked to
be on the mailing list.
